# Environmental control of mammary carcinoma cell expansion by acidification and spheroid formation in vitro

**DOI:** 10.1038/s41598-020-78989-6

**Published:** 2020-12-15

**Authors:** Ana Carolina Lima Ralph, Iuri Cordeiro Valadão, Elaine Cristina Cardoso, Vilma Regina Martins, Luanda Mara Silva Oliveira, Estela Maris Andrade Forell Bevilacqua, Murilo Vieira Geraldo, Ruy Gastaldoni Jaeger, Gary S. Goldberg, Vanessa Morais Freitas

**Affiliations:** 1 Department of Cell and Developmental Biology, Institute of Biomedical Sciences, University of São Paulo, São Paulo, Brazil; 2 International Research Center, A. C. Camargo Cancer Center, National Institute of Science and Technology in Oncogenomics & Therapeutic Innovation, São Paulo, Brazil; 3 Laboratory of Dermatology and Immunodeficiencies (LIM-56), Department of Dermatology, Institute of Tropical Medicine, University of São Paulo Medical School, São Paulo, Brazil; 4 Department of Structural and Functional Biology, Institute of Biology, State University of Campinas, Campinas, Brazil; 5 Graduate School of Biomedical Sciences and Department of Molecular Biology, Rowan University School of Osteopathic Medicine, Stratford, NJ USA

**Keywords:** Cell biology, Cancer, Breast cancer, Cancer microenvironment

## Abstract

Breast cancer is the leading cause of cancer death among women worldwide. Like other cancers, mammary carcinoma progression involves acidification of the tumor microenvironment, which is an important factor for cancer detection and treatment strategies. However, the effects of acidity on mammary carcinoma cell morphology and phenotype have not been thoroughly characterized. Here, we evaluated fundamental effects of environmental acidification on mammary carcinoma cells in standard two-dimensional cultures and three-dimensional spheroids. Acidification decreased overall mammary carcinoma cell viability, while increasing their resistance to the anthracycline doxorubicin. Environmental acidification also increased extracellular vesicle production by mammary carcinoma cells. Conditioned media containing these vesicles appeared to increase fibroblast motility. Acidification also increased mammary carcinoma cell motility when cultured with fibroblasts in spheroids. Taken together, results from this study suggest that environmental acidification induces drug resistance and extracellular vesicle production by mammary carcinoma cells that promote tumor expansion.

## Introduction

Breast cancer accounts for 30% of female cancers, and is the most common cause of cancer death among women worldwide^1^. Breast cancer treatment usually involves surgery in concert with chemotherapy and radiation according to tumor characteristics^2^. The average 5-year survival rate for women with invasive breast cancer is over 90%, but less than 30% if the cancer has metastasized to other sites in the body^1,2^.

In general, cancer aggression relies on tumor cell proliferation leading to increased glucose consumption and cellular competition for limited nutrients, followed by metabolic reprogramming to enhance glycolysis (called the “Warburg effect”), which causes chronic acidification of the tissue microenvironment^3^. Environmental acidification, imposes selection pressures that cause genomic instability^4^, VEGF production leading to angiogenesis^5^ and immunomodulation^6^ that increase tumor heterogeneity and aggression^7,8^. This has led to the development of drug delivery strategies based on acidic environmental conditions including proton pump inhibitors, proton-sensing G protein-coupled receptors, and cytotoxic agents activated at low pH^9–11^.

Environmental changes can trigger a cell signaling mechanisms including the production of extracellular vesicles. These particles are released from cells and contain a variety of molecules including proteins, lipids, and nucleic acids that affect normal and transformed cell growth and morphology^12–14^. Extracellular vesicles have emerged as diagnostic and prognostic biomarkers for conditions including cardiovascular disease^15^, Alzheimer’s disease^16^, and breast cancer^17^. Environment acidity has been found to increase extracellular vesicle production in several human cancer cell types^18^. The reduced pH of the tumor tissues provides a hostile microenvironment that transform cells can adapt to as they produce extracellular vesicles^19,20^. These vesicles provide a unique mechanism for intercellular communication that promotes transformed cell growth^21^, immune tolerance^22^, and drug resistance^23^.

While the importance of hypoxia, metabolic programming reprogramming, and acidosis on cancer progression is clearly evident, the influence of acidity on tumor-stromal cell interactions and motility have not been thoroughly characterized. In this study, the effects of environmental acidification were examined on mammary carcinoma cells in standard two-dimensional cultures and three-dimensional spheroids including fibroblasts. While acidification decreased overall mammary carcinoma cell viability, it increased the resistance of these cells to doxorubicin exposure. Environmental acidification also increased the production of extracellular vesicles by mammary carcinoma cells, and conditioned medium containing these vesicles appeared to increase fibroblast motility. Moreover, acidification increased mammary carcinoma cell motility when cultured with fibroblasts in spheroids.

## Materials and methods

### Cell lines and culture conditions

MCF7, MDA-MB-231, and T-47D human mammary adenocarcinoma cells were cultured in Dulbecco’s Modified Eagle’s Medium (DMEM, Sigma Chemical Co., St. Louis, MO, USA). Nontransformed human fibroblasts were kindly provided by Dr. Silvya Stuchi Maria-Engler (School of Pharmaceutical Sciences FCF-USP), and NTera-2 was kindly provided by Dr. Rodrigo Alexandre Panepucci (Fundação Hemocentro de Ribeirão Preto, FUNDHERP). Nontransformed skin fibroblasts and human embryonal carcinoma cell line NTera-2 were cultured with DMEM-F12 (Sigma). While mammary carcinoma fibroblasts offer specific advantages including production of mammary gland extracellular matrix proteins that affect gene expression which regulates mammary epithelial cell proliferation, differentiation, macrophage recruitment, and angiogenesis^24–27^, skin fibroblasts were used here to study the influence of stroma-tumor interactions^28^. We have used these cells to study interactions including extracellular vesicle communication between fibroblasts and mammary carcinoma cells^29^. This strategy offers standardization and mechanistic reproducibility evidenced by suggestion for use with patient derived tumor cells for personalized cancer patient care^30^. All cell lines were maintained at 37 °C in 5% CO_2_ with 100% humidity. All media were supplemented with 10% Fetal Bovine Serum (FBS, Gibco, Life Technologies, Grand Carlsbad, CA) and antibiotics (100 U/mL penicillin, 100 µg/mL streptomycin; Gibco). Acidic media was prepared by addition of 5 N HCl to achieve desired pH followed by filter sterilization. Fibroblast were isolated with approval of the Local Ethics Committee HU CEP case Number 943/09 and CEP FCF/USP 534.

### Cell viability assays

MTT (3-(4,5-dimethylthiazol-2-yl)-2,5-diphenyltetrazolium bromide) assay was performed as previously described^31^. Cells (1.0 × 10^4^ cells/well) were incubated in media with indicated pH (range pH 6.0–7.2) for 24 h. MTT (5.0 mg/mL) was then added and formazan crystals were dissolved in DMSO for absorbance measured at 570 nm using an Epoch Microplate Spectrophotometer (BioTek, Winooski, Vermont, USA).

### Fluorescence-based morphological analysis

Cells (1.0 × 10^4^) were allowed to attach on sterile coverslips for 24 h in standard media (pH 7.2). Media was then changed to media with pH 6.2 or 7.2, and cells were incubated for an additional 24 h. Cells were then fixed with 4% formaldehyde/PBS for 10 min and permeabilized with 0.5% Triton X-100/PBS (Sigma) for 10 min. Cells were then incubated with phalloidin 1:500 conjugated to Alexa Fluor 488 (Life Technologies) for 1 h protected from light and mounted in ProLong with DAPI (Life Technologies) for nuclei staining.

### Transmission electron microscopy (TEM) analysis

Cells were grown to 90% confluence before being washed, scraped, pelleted, and fixed in 2.5% glutaraldehyde in 0.1 M sodium cacodylate buffer (pH 7.2) overnight. Samples were post fixed in 1% osmium tetroxide for 2 h, stained in bloc with 0.5% uranyl acetate, rinsed, and dehydrated in graded ethanol. After immersion in propylene oxide, samples were embedded in epoxy resin (Spurr, Electron Microscopy Sciences, EMS, Hatfield PA, USA) and polymerized for 42 h at 75 °C. Ultrathin sections were stained with lead citrate and uranyl acetate and examined in a FEI Tecnai G20 Electron microscope at 200 kVA.

### Cell migration assay

Cells were transferred to wells containing inserts (Ibidi, Munich, Germany). Inserts were removed after 24 h leaving confluent monolayers with a defined cell-free gap of 500 μm. Alternatively, a line was drawn in confluent monolayers by a P10 pipet tip. Cells were then washed twice with PBS, and incubated in media with pH 7.2 or pH 6.2. Wound closure was monitored and imaged by standard microscopy at 0, 24, and/or 48 h. Data were quantitated as the percentage the wound filled with cells relative to time 0.

### Spheroid formation and analysis

Spheroids were formed by the hanging drop method as previously described^32^. Briefly, MCF7 cells were suspended at a concentration of 1 × 10^6^ cell/ml in spheroid medium (complete medium at pH 7.2 or 6.2 supplemented with 1.2% w/v methylcellulose), and 20 μl (2000 cells) were pipetted onto lids of 100 mm dishes containing PBS. Cells were incubated for 24 h, and resulting spheroids were imaged by phase contrast microscopy and analyzed by ImageJ software. To examine spheroid expansion, MCF7 cells were incubated at pH 7.2 or 6.2 for 24 h and labeled for 30 min with Cell Tracker Green CMFDA, AM (Life Technologies), or Cell Tracker orange CMRA, AM, (Life Technologies) if cultured with fibroblasts labeled with Cell Tracker Green. Labeled cells were washed with PBS, and incubated in complete medium for an additional 3 h at appropriate pH. Labeled MCF7 cells or fibroblasts were then trypsinized and suspended in spheroid medium at 1 × 10^5^ cells/ml or 5 × 10^4^ cells/ml, respectively. After 24 h growth on lids, spheroids were transferred to a glass coverslip coated with 0.1% gelatin, and allowed to adhere in 500 µL complete medium for 2 h. After 0, 24, and/or 48 h, spheroids were fixed with 8% PFA for 15 min and imaged by confocal microscopy on a Leica TCS AOBS SP8 Tandem Scanner with a spectral Leica SP detection system (Leica Microsystems, Germany). Spheroid expansion relative to the initial 0 h time point was measured using ImageJ software.

### Extracellular vesicles analysis

Cells were cultured to confluence in 6-well plates, washed, and cultured in serum-free medium (1 ml/well) at pH 7.2 or pH 6.2 for 24 h. Conditioned media (CM) was then collected, centrifuged 15,000*g* for 30 min at 4ºC to remove cells and other debris, and stored at – 80 °C until analysis by NTA (Nanoparticle Tracking Analysis) in a NanoSight LM10 system (Amesbury, UK) equipped with NTA 3.0 software for particle size calculations. Acquisitions were performed in three records of 60 s with specified shutter (604), gain (100), and threshold (10) values. Cells were counted after CM removal to calculate vesicles produced per cell.

## Results

### Acidic pH exposure reduces MCF7 mammary carcinoma cell viability, proliferation, and migration

Mammary carcinoma evolves from benign or premalignant cells that evolve into aggressive malignancies. For example, MCF7 cells are moderately differentiated mammary carcinoma cells with a relatively low invasive and metastatic potential. However, these cells respond to selective pressures that alter their morphology and neoplastic phenotype. These processes can be controlled in culture to mimic neoplastic alterations that promote breast cancer progression in vivo. MCF7 cells respond to tumorigenic estrogen and progesterone signaling events that are relatively well studied^33^. These cells also respond to environmental cues including TRAIL production during anoikis^34^ and contact normalization mediated by junctional communication with surrounding nontransformed cells^35^. In contrast, mechanisms by which environmental stress including the Warburg effect lead to adaptive responses that promote tumor progression are not clearly defined. In particular, effects of pH acidification in the tumor microenvironment during this process, and how these effects increase mammary carcinoma invasion and metastatic potential remain to be elucidated. MCF7 cells are well suited to serve as a model on which to examine how environmental acidity induces morphological and phenotypic changes that affect mammary carcinoma cell growth and invasion^33^.

**Figure 1:**
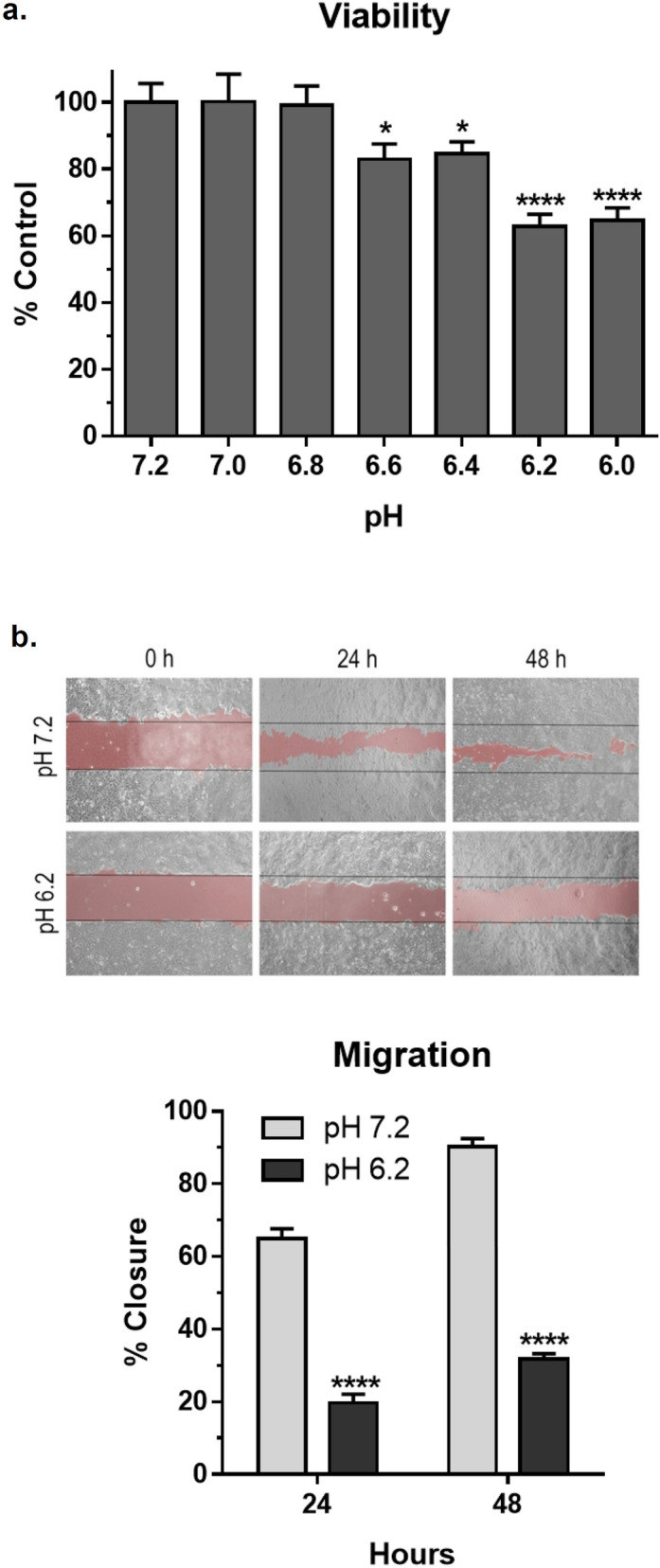
Acidic pH decreases MCF 7 mammary carcinoma cell viability and proliferation. (**a**) Cell viability was examined by MTT assay after 24 h growth at indicated pH. Data are shown as percent of control cells grown at pH 7.2 (mean + SEM, n = 8). Single and quadruple asterisks indicated p < 0.05 and p < 0.0001 compared to control cells by t-test, respectively. (**b**) Cell migration was evaluated by wound healing during 24 and 48 h growth at pH 7.2 or pH 6.2. Phase-contrast images are presented along with data shown as the percent of the wound that was filled by cells (mean + SEM, n = 4) with quadruple asterisks indicating p < 0.0001 by t-test.

Mammary carcinoma tumor microenvironments display pH values ranging between an acidic value of 6.0 to a normal physiological mammary epithelial pH of 7.2^36^. MCF7 cells cultured for 24 h in this pH range indicated an acute cytotoxic effect in response to this environmental acidification. Cell viability and proliferation were both decreased by over 30% at pH 6.2 compared to pH 7.2 as shown in Fig. 1a,b, respectively. This acidic pH 6.2 value had an acute effect on MCF7 cell growth, and is consistent with mammary carcinoma microenvironments in vivo. Therefore, pH 6.2 was chosen to contrast the effects of acidity on these cells with that of normal physiological pH 7.2.

In addition to inhibiting cell growth, pH acidity also decreased MCF7 cell migration in standard cell culture. MCF7 cells cultured at pH 6.2 migrated over twofold less than cells cultured at pH 7.2 as shown in Fig. 1c. These data indicate that microenvironmental acidification causes acute effects that decrease mammary carcinoma cell growth and motility.

### Acidic pH induces morphological changes and increases drug resistance of MCF7 mammary carcinoma cells

**Figure 2:**
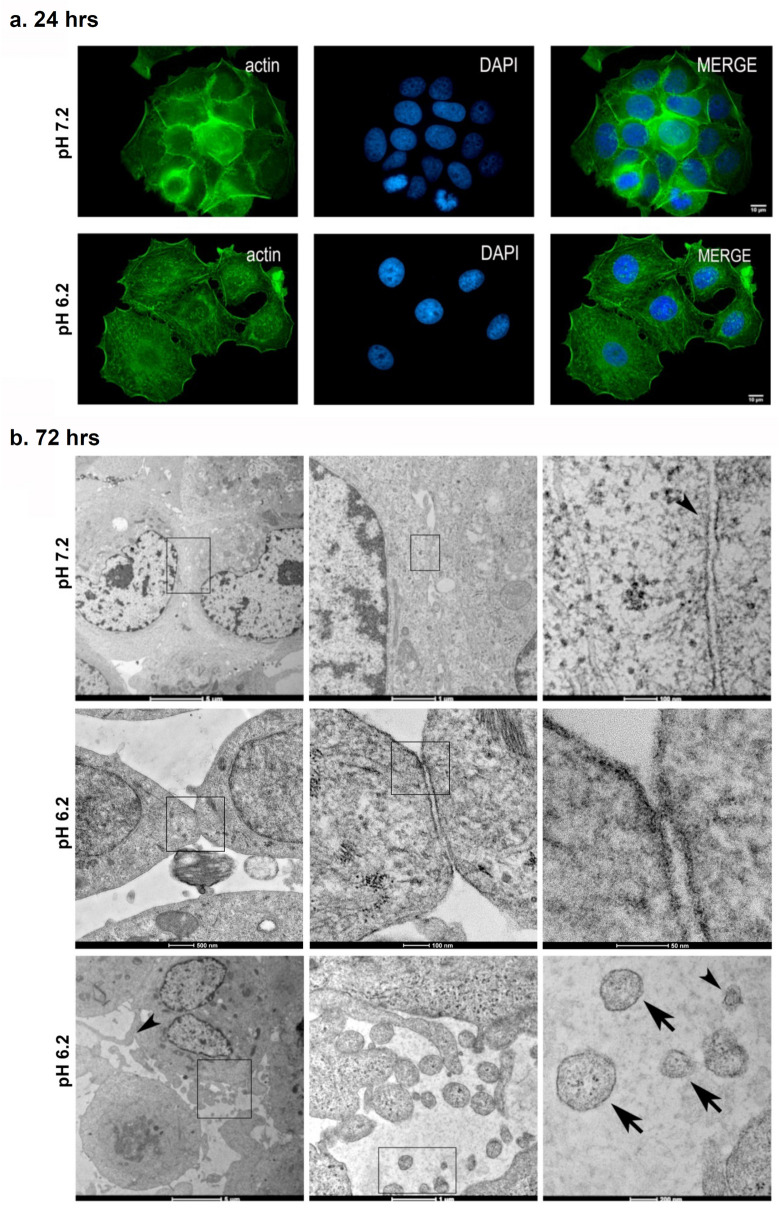
Acidic pH disrupts MCF 7 mammary carcinoma intercellular junctions and induces extracellular vesicle formation. (**a**) Cytoplasmic actin and nuclei were visualized by fluorescence staining with Phalloidin-Alexa 488 (green) and DAPI (blue) after 24 h growth at pH 7.2 or 6.2 and visualized by confocal microscopy as indicated. (**b**) Cell morphology was examined by transmission electron microscopy after 72 h growth at pH 7.2 or 6.2 as indicated. Cells grown at pH 7.2 display normal intercellular junctions (arrowheads). In contrast, cells grown at pH 6.2 display distinct heterochromatin disposition and wider spaces between cells (arrowheads), as well as structures suggestive of microvesicles (arrows) and exosomes (arrowhead). Boxes in each image indicate progression magnifications moving from left to right hand panels.

MCF7 cells in pH 7.2 displayed a cobblestone-like morphology with notable intercellular contacts evident of a pronounced epithelial phenotype. In contrast, cells growth for 24 h at pH 6.2 displayed notable loss of intercellular contact and increased numbers of isolated cells. Cells grown for 24 h at pH 6.2 also displayed a decrease in cortical actin fibers along with an increase in actin stress fibers compared to cells grown at pH 7.2 visualized by fluorescence microscopy as shown in Fig. 2a. Transmission electron microscopy analysis of MCF7 cells grown for 72 h confirmed these findings. Cells cultured at pH 6.2 displayed distinct heterochromatin disposition, and modifications in shape with consequential decreases in intercellular junctions compared to cells growth at pH 7.2 as shown in Fig. 2b. Additionally, cells grown at pH 6.2 presented extracellular vesicles indicative of exosomes and microvesicles that were not evident in cells growth at pH 7.2 (see arrows in Fig. 2b).

**Figure 3:**
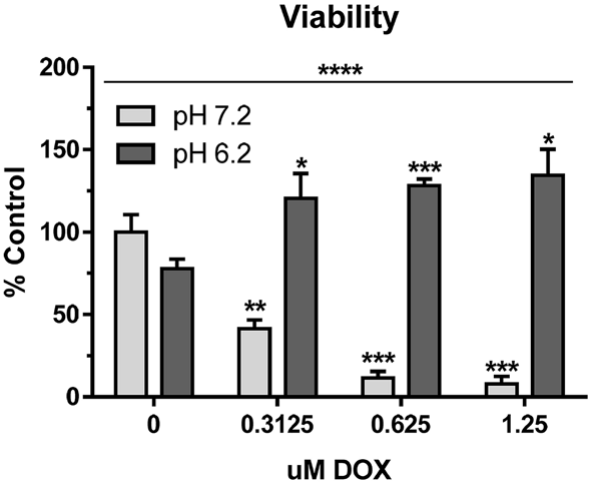
Acidic pH decreases MCF 7 increases doxorubicin drug resistance. Cell viability was examined by MTT assay after 72 h growth at indicated pH 6.2 or pH 7.2 in concentrations of doxorubicin as indicated. Data are shown as percent of control cells grown at pH 7.2 (mean + SEM, n = 4) with quadruple asterisks indicating p < 0.0001 by ANOVA, and single, double, and triple asterisks indicating p < 0.05, 0.01, and 0.001 by t-test compared to untreated controls, respectively as indicated.

Morphological changes at 72 h seen in Fig. 2b suggest that mammary carcinoma cells undergo an adaptive response to chronic acidity. Microenvironmental acidification and ECM alterations implicated in chemoresistance to drugs such as doxorubicin^37,38^. Accordingly, MCF7 cells grown for 72 h at pH 6.2 displayed several time more growth than cells growth at pH 7.2 when treated with doxorubicin. These effects were evident in a dose dependent manner along physiologically relevant concentrations of doxorubicin ranging from 0.3 to 1.25 µM^39^ as shown in Fig. 3.

### MCF7 mammary carcinoma cells form expanding spheroids in neutral and acidic environments

**Figure 4:**
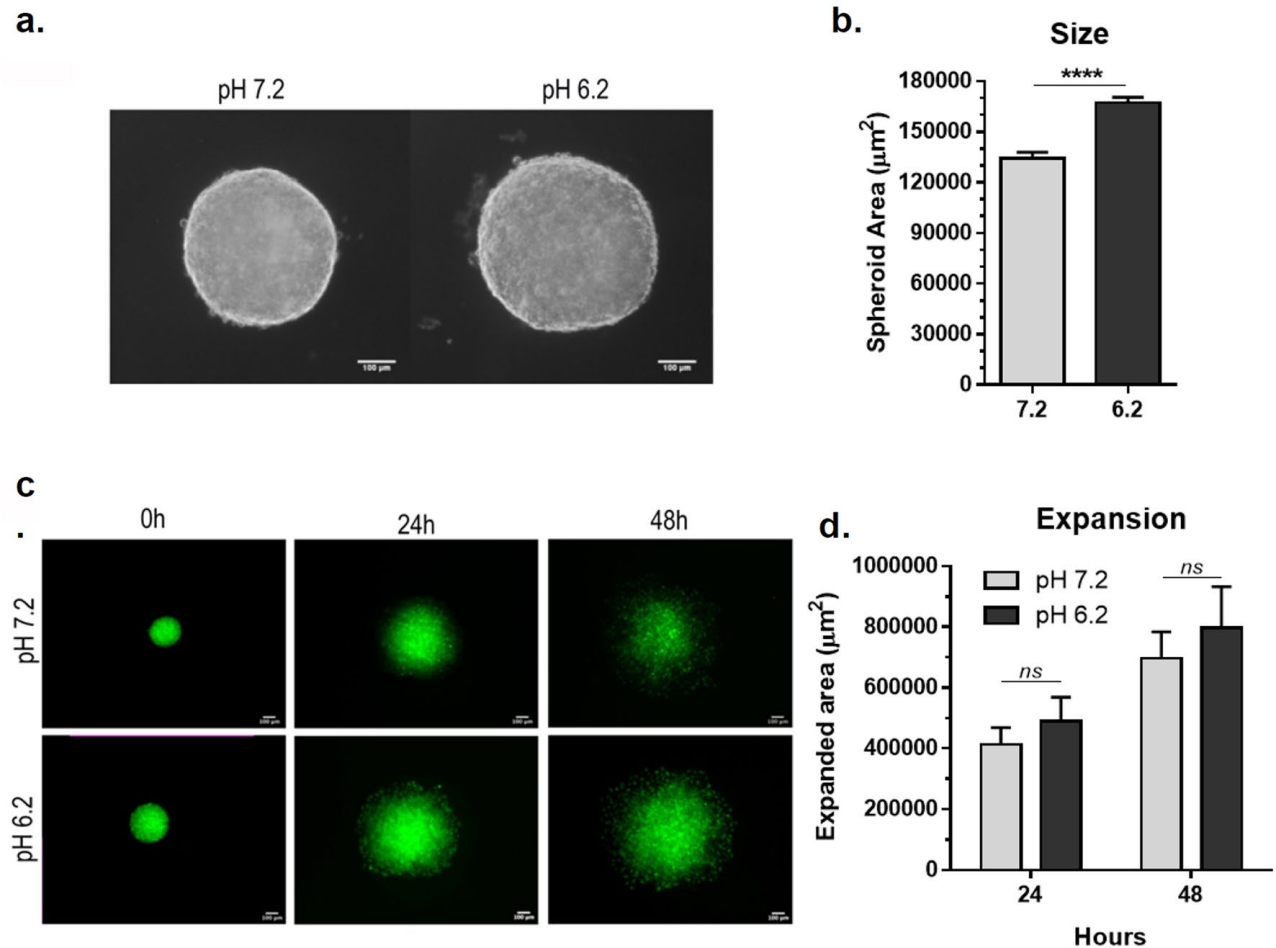
MCF7 mammary carcinoma spheroid expansion occurs in neutral and acidic environments. (**a**) MCF7 spheroids formed by the hanging drop method were imaged by phase-contrast microcopy after 24 h growth at pH 7.2 or 6.2 as indicated. (**b**) Spheroid area was measured and shown as µm^3^ (mean + SEM, n = 10). (**c**) MCF7 spheroids labeled with Cell Tracker Green were incubated on glass coverslip coated with gelatin at pH 7.2 or 6.2 and imaged by confocal microscopy at indicated time points. (**d**) Spheroid expansion relative to the initial 0 h time point was measured and shown as µm^2^ (mean + SEM, n = 5). Quadruple asterisks and ns indicate p < 0.001 and > 0.05 by t-test as indicated.

MCF7 cells retain the ability to generate 3 dimensional spheroids similar to nontransformed mammary epithelial cells. This property can be used to examine tumor progression of these cells in a situation more similar to in vivo than standard growth on cell culture dishes^33^. We utilized a hanging drop assay^32^ to investigate the effects of pH on these mammary carcinoma cells in spheroid formation. In contrast to cell growth in standard culture (see Fig. 1), low pH did not inhibit the ability of MCF7 cells to form spheroids. Indeed, spheroids grew significantly better at pH 6.2 than pH 7.2 as shown in Fig. 4a,b.

MCF7 cells were fluorescently labeled, and resulting spheroids were placed onto gelatin coated glass surfaces to examine outward expansion by confocal microscopy. These conditions found that spheroid expansion was not inhibited by acidic pH. As shown in Fig. 4c,d, these cells expanded in pH 6.2 at least as well as they did at pH 7.2.

### Acidic pH augments mammary MCF7 carcinoma cell extracellular vesicle production

**Figure 5:**
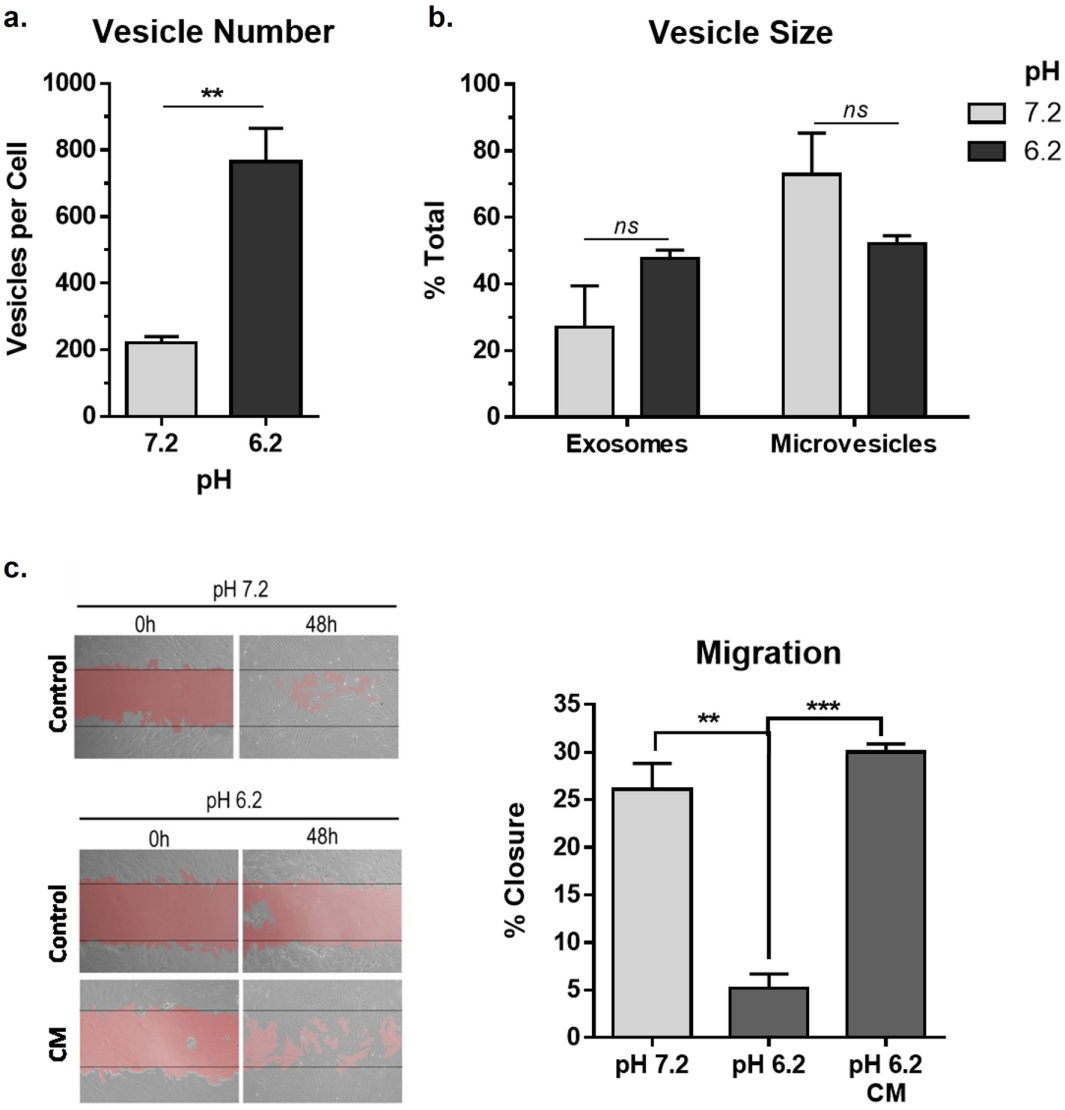
Acidic pH augments MCF7 mammary carcinoma cell extracellular vesicle production. (**a**) Extracellular vesicles produced by MCF7 cells cultured for 24 h at pH 7.2 or 6.2 were analyzed by NanoSight tracking analysis and shown as vesicles per cell (mean + SEM, n = 3). (**b**) Vesicle size was measured, grouped into exosomes (30–110 nm) or microvesicles (> 110 nm), and shown as percent of the total number of vesicles (mean + SEM, n = 3). Double asterisks indicate p < 0.01 by t-test. (**c**) Cell migration was examined in fibroblasts cultured in serum free media with or without addition of conditioned media containing exosomes from MCF7 cells cultured for 24 h at pH 6.2 as indicated. Phase-contrast images are presented along with data shown as the percent of the wound that was filled by cells (mean + SEM, n = 3) with ns, double, and triple asterisks indicating p > 0.05, p < 0.01 and p < 0.001 by t-test, respectively.

Extracellular vesicles have emerged as important signaling modulators that promote mammary carcinoma cell motility, invasion, and tumor progression^29,40,41^. Environmental acidosis enhanced extracellular vesicle production seen by electron microscopy as shown in Fig. 2. This observation was verified by NanoSight tracking analysis (NTA). MCF7 cells produced an nearly four times as many extracellular vesicles at pH 6.2 (765 ± 100 particles per cell) than at pH 7.2 (221 ± 19 particles per cell) over 24 h as shown in Fig. 5a. Particles the size of exosomes (30–110 nm) and microvesicles (> 110 µm) were produced at both pH conditions as shown in Fig. 5b.

Conditioned media containing extracellular vesicles produced by MCF7 cells was added to fibroblasts grown at pH 6.2 to evaluate its effects on cell motility. Fibroblasts cultured in serum free medium at pH 7.2 migrated about fivefold more than they did at pH 6.2. However, fibroblasts migrated equally well when medium was replaced with serum free medium conditioned for 24 h by MCF7 cells as shown in Fig. 5c. These data suggest that microvesicles or other factors produced by MCF7 cells significantly enhanced fibroblast motility.

### Acidic pH enhances movement of MCF7 cells cultured in spheroids with fibroblasts

**Figure 6:**
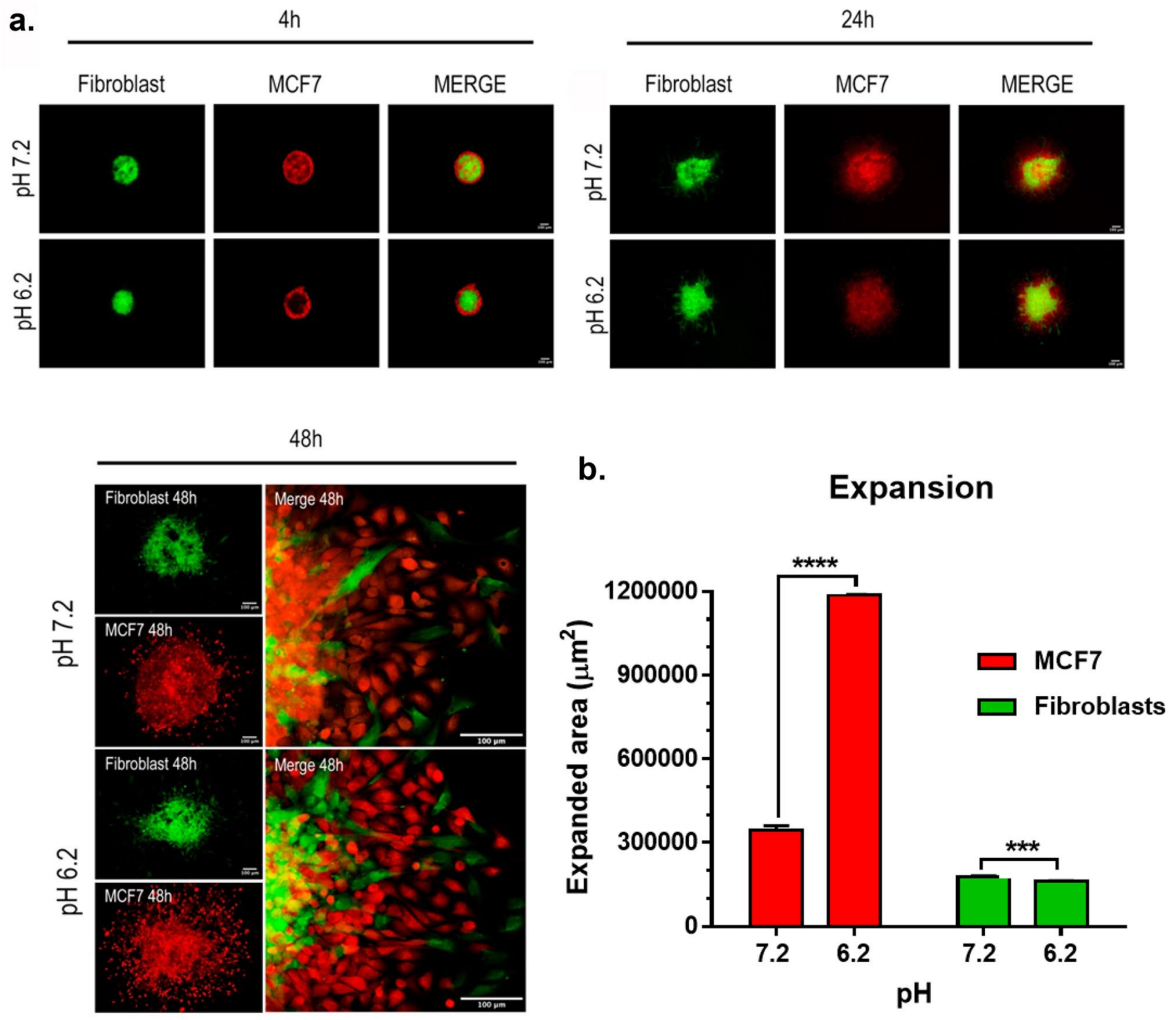
Acidic pH enhances MCF7 cell migration in spheroid cocultures with fibroblasts. (**a**) Fluorescently labeled fibroblasts (green) and MCF7 cells (red) were cultured together in spheroids and visualized by confocal microscopy at 4, 24, and 48 h of growth at pH 7.2 or 6.2 as indicated. (**b**) Fibroblast and MCF7 cell motility from spheroid expansion relative to the initial time point was measured and shown as µm^2^ (mean + SEM, n = 5). Triple and quadruple asterisks indicate p < 0.001 and p < 0.0001 by t-test, respectively.

Interactions between mammary carcinoma cells and neighboring fibroblasts are important modulators of malignant expansion. We cultured MCF7 cells with fibroblasts in spheroids to investigate the effects of pH on cell motility in this environment. Fibroblasts and MCF7 cells were fluorescently labeled green and red, respectively, to track their movement in these cultures. Cells were visualized by confocal microscopy at 4 h, 24 h, and 48 h of growth at pH 7.2 or pH 6.2 as shown in Fig. 6.

Interactions between fibroblasts and MCF7 cells orchestrated an interesting space configuration by 4 h of growth. MCF7 cells surrounded fibroblasts in the core of the spheroids at this time point. This configuration persisted for at least 24 h as shown in Fig. 6a. This effect seen at both pH values, but seemed more evident at pH 6.2 than at pH 7.2. MCF7 cells appeared to invade the center of these spheroids along with fibroblasts more at pH 7.2 than at pH 6.2 (see 24 h time point in Fig. 6a).

Fibroblast and MCF7 cell migration were measured as expansion from the spheroids at 48 h of growth as shown in Fig. 6. MCF7 spheroids migrated over threefold more in pH 6.2 than at pH 7.2. In contrast, fibroblasts migrated about 10% more in pH 7.2 than at pH 6.2 (see Fig. 6b). These data indicate that acidic pH preferentially enhances MCF7 cell expansion when cultured with fibroblasts in spheroids.

### Acidic pH does not significantly affect MDA-MB-231 mammary carcinoma cell viability or extracellular vesicle production

**Figure 7:**
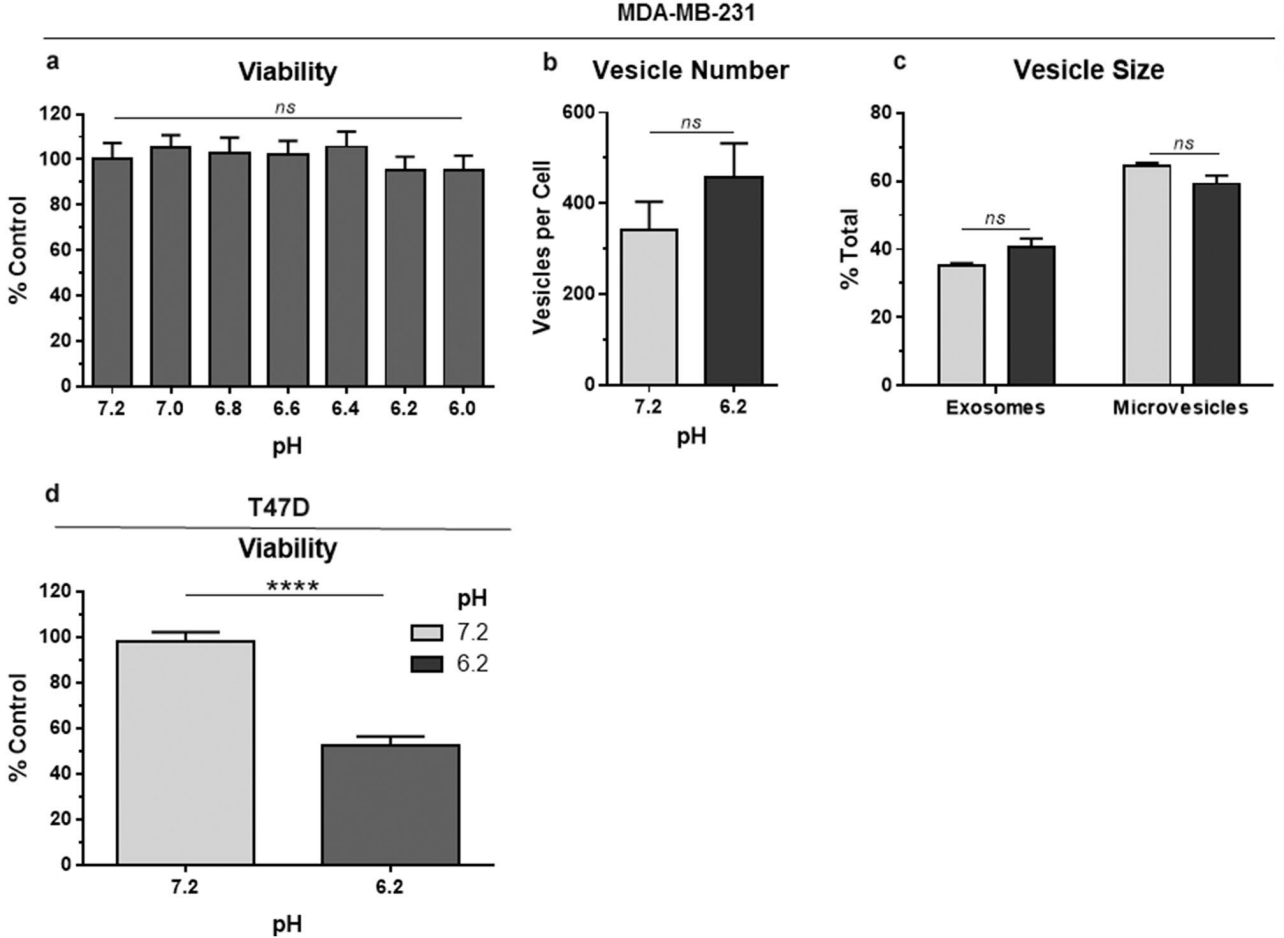
Acidic pH does not significantly affect MDA-MB-231 mammary carcinoma cell viability or extracellular vesicle production. (**a**) Viability of MDA-MB-231 or (**d**) T-47D cells was examined by MTT assay after 24 h growth at indicated pH. Data are shown as percent of control cells grown at pH 7.2 (mean + SEM, n = 8). (**b**) Extracellular vesicles produced by MDA-MB-231 cells cultured for 24 h at pH 7.2 or 6.2 were analyzed by NanoSight tracking analysis and shown as vesicles per cell (mean + SEM, n = 6). (**c**) Vesicle size was measured, grouped into exosomes (30–110 nm) or microvesicles (> 110 nm), and shown as percent of the total number of vesicles (mean + SEM, n = 3). ns indicates p > 0.05 as indicated by t-test.

Effects of environmental acidification on viability of MDA-MB-231 cells were examined to compare their response with less aggressive MCF7 cells. MDA-MB-231 are highly tumorigenic triple negative mammary carcinoma cells^35,42^. In contrast with MCF7 cells which exhibited a decrease in viability of over 30% at pH 6.2 compared to pH 7.2 (see Fig. 1a), reduced pH did not significantly affect MDA-MB-231 cell viability as shown in Fig. 7a. T-47D cells were employed to confirm the role of nonaggressive phenotype on the effect of acidification on mammary carcinoma cell viability^43^. Like MCF7 cells, these nonaggressive cells exhibited a significant decrease in viability at pH 6.2 compared to pH 7.2 as shown in Fig. 7b.

In addition to viability, environmental acidification did not increase extracellular vesicle production by MDA-MB-231 cells. In contrast to MCF7 cells which produced about 4 times more extracellular vesicles at pH 6.2 than pH 7.2, MDA-MB-231 cells produced about 400 vesicles per cells at either pH as shown in Fig. 7c. This was more than the approximately 200 vesicles per cells produced by MCF7 cells at pH 7.2, but less than the approximately 750 vesicles per cell produced by MCF7 cells at pH 6.2. In either case, there seemed to be a general trend to produce more exosomes and less microvesicles at pH 6.2 than pH 7.2 as shown in Fig. 7d.

## Discussion

Cancers are composed of heterogeneous, plastic, and metabolically complex populations of cells adapted to abnormal microenvironmental conditions^44^. In particular, tumors are often characterized by a more acid extracellular pH than corresponding normal tissues. Most human tumors exhibit a range of pH values between 5.85 and 7.68, while normal tissues usually have pH values between 7.0 and 7.4^45^. Therefore, acidosis is a typical characteristic of the tumor microenvironment. Acidic pH ranges (6.0–6.8) have been used as adjuvant for cancer probes to detect and diagnose cancer tissues including mammary carcinoma^36,46^. Mammary carcinoma tumor microenvironments display pH values ranging between an acidic value of 6.0 to a normal physiological value of 7.2^36^.

MCF7 cells were used here to represent mammary carcinoma cells with the potential to develop aggressive phenotype properties in response to environmental acidification^33^. We report here that the viability and motility of MCF7 cells was reduced by over 30% and 50% at pH 6.2 compared at pH 7.2 in standard cell culture. These effects of environmental acidification corroborate previous reports^47–49^, and may be due to the impact of acid-basic transporters on cell cycle progression acting as cell viability regulators. For example, knockdown of either NHE1 or NBCn1 caused a significant delay cell cycle progression in MCF7 cells^50,51^.

Acidification at pH 6.2 serves an important factor for extracellular imaging agents and drugs that anchor to the plasma membrane of cancer cells^52^. Microenvironmental acidification also triggers chemoresistance to drugs such as doxorubicin^37,38^. Accordingly, we found in this study that MCF7 cells grown for 72 h at pH 6.2 were significantly more resistant to doxorubicin than cells grown at pH 7.2. These effects were dose dependent along physiologically relevant concentrations of doxorubicin^39^.

Tumor-secreted extracellular vesicles apply complex effects on local stromal or distant microenvironments. They contain bioactive molecules, such as nucleic acids, proteins and lipids that can influence the function of the recipient cell^53^. Acidification has been reported to increase cancer cell exosome production^19,54^. These extracellular vesicles are derived from endosomes as opposed to ectosomes derived from plasma membrane or apoptotic bodies^12,55^.

In this study, we found that environmental acidification of MCF7 cells caused morphological changes and the production of extracellular vesicles including exosomes and microvesicles. Conditioned medium containing these extracellular vesicles appeared to enhance tumor cell motility. In addition to extracellular vesicles, MCF7 cells can secrete factors that promote angiogenesis and cell motility^17,56^. Therefore, diffusible factors in conditioned media might have contributed to the effects of acidification on mammary carcinoma cells described in this study. However, mammary carcinoma cells have been reported to produce extracellular vesicles that promote fibroblast cell migration^29^. This crosstalk between cancer cells and stroma plays an important role in cancer progression^57^.

Both MDA-MB-231 and MCF7 cells have been reported to display doxorubicin resistance accompanied by loss of E-cadherin junctions along with production of N-cadherins in response to environmental acidification and growth in spheroid cultures^42,58,59^. Our data indicate that effects of pH on cell viability and extracellular vesicle production are more pronounced by less aggressive MCF7 than more aggressive triple negative MDA-MB-231 cells. However, the role of cadherins and epithelial mesenchymal transition in this process has not been clearly defined or addressed in the present study. Nonetheless, the effects of environmental acidification and spheroid structures on tumor cell adaptation are clearly evident.

Multicellular spheroids can be used to investigate complexities of the avascular tumor microenvironment^51,60^. Spheroids containing fibroblasts and MCF7 cells can be used to investigate how interactions between stromal and carcinoma cells control tumor growth and expansion^61^. We cultured MCF7 cells with fibroblasts in spheroids to investigate how pH affects these interactions and cell motility. Initially, MCF7 cells surrounded fibroblasts in the core of these spheroids. Environmental acidification enhances motility of MCF7 cells into the body of these spheroids, and also enhances their expansion to extend the growth of these structures by over threefold. These results are corroborated by reports of differences tumor cell migration in 3 dimensional versus 2 dimensional configurations^62^. Taken together, results from this investigation indicate that environmental acidification induces changes in intercellular architecture that promote mammary carcinoma cell chemoresistance, and the production of extracellular vesicles and possibly other diffusible factors to increase tumor cell motility.

## Data Availability

All data generated and reported from this study are included in this article.
